# Prediction of mortality in hospitalized Egyptian patients with Coronavirus disease-2019: A multicenter retrospective study

**DOI:** 10.1371/journal.pone.0262348

**Published:** 2022-01-11

**Authors:** Muhammad M. AbdelGhaffar, Dalia Omran, Ahmed Elgebaly, Eshak I. Bahbah, Shimaa Afify, Mohamed AlSoda, Mohamed El-Shiekh, Enass S. ElSayed, Soha S. Shaaban, Samah AbdelHafez, Khaled Elkelany, Ayman A. Eltayar, Omnia S. Ali, Lamiaa Kamal, Ahmed M. Heiba, Ahmad El Askary, Hend Ibrahim Shousha

**Affiliations:** 1 Gastroenterology & Infectious Diseases Department, Ahmed Maher Teaching Hospital, Cairo, Egypt; 2 General Organization for Teaching Hospitals and Institutes (GOTHI), Cairo, Egypt; 3 Endemic medicine department, Faculty of Medicine, Cairo University, Cairo, Egypt; 4 Faculty of Medicine, Al-Azhar University, Cairo, Egypt; 5 Gastroenterology Department, National Hepatology and Tropical medicine Research Institute, Cairo, Egypt; 6 Chest unit, Matareya Teaching Hospital, Cairo, Egypt; 7 Nephrology Department, Ahmed Maher Teaching Hospital, Cairo, Egypt; 8 Rheumatology department, El Matareya Educational Hospital, Cairo, Egypt; 9 Pediatric departement, Shebin Elkom Teaching Hospital, Shebin Elkom, Egypt; 10 Intensive care Department, Damanhour Teaching Hospital, Damanhour, Egypt; 11 Clinical and chemical pathology department, Ahmed Maher Teaching Hospital, Cairo, Egypt; 12 Clinical and chemical pathology department, Sahel General Hospital, Cairo, Egypt; 13 Internal Medicine Department, National Research Centre, Cairo, Egypt; 14 Department of Clinical Laboratory Sciences, College of Applied Medical Sciences, Taif University, Taif, Saudi Arabia; Post Graduate Institute of Medical Education and Research, INDIA

## Abstract

We aimed to assess the epidemiological, clinical, and laboratory characteristics associated with mortality among hospitalized Egyptian patients with COVID-19. A multicenter, retrospective study was conducted on all polymerase chain reaction (PCR)-confirmed COVID-19 cases admitted through the period from April to July 2020. A generalized linear model was reconstructed with covariates based on predictor’s statistical significance and clinically relevance. The odds ratio (OR) was calculated by using stepwise logistic regression modeling. A total of 3712 hospitalized patients were included; of them, 900 deaths were recorded (24.2%). Compared to survived patients, non-survived patients were more likely to be older than 60 years (65.7%), males (53.6%) diabetic (37.6%), hypertensive (37.2%), and had chronic renal insufficiency (9%). Non-survived patients were less likely to receive azithromycin (p <0.001), anticoagulants (p <0.001), and steroids (p <0.001). We found that age ≥ 60 years old (OR = 2.82, 95% CI 2.05–3.86; p <0.0001), diabetes mellitus (OR = 1.58, 95% CI 1.14–2.19; p = 0.006), hypertension (OR = 1.69, 95% CI 1.22–2.36; p = 0.002), chronic renal insufficiency (OR = 3.15, 95% CI 1.84–5.38; p <0.0001), tachycardia (OR = 1.65, 95% CI 1.22–2.23; p <0.001), hypoxemia (OR = 5.69, 95% CI 4.05–7.98; p <0.0001), GCS <13 (OR 515.2, 95% CI 148.5–1786.9; p <0.0001), the use of therapeutic dose of anticoagulation (OR = 0.4, 95% CI 0.22–0.74, p = 0.003) and azithromycin (OR = 0.16, 95% CI 0.09–0.26; p <0.0001) were independent negative predictors of mortality. In conclusion, age >60 years, comorbidities, tachycardia, hypoxemia, and altered consciousness level are independent predictors of mortality among Egyptian hospitalized patients with COVID-19. On the other hand, the use of anticoagulants and azithromycin is associated with reduced mortality.

## Introduction

The clinical features and outcomes of Coronavirus disease-2019 (COVID-19) vary substantially from asymptomatic/mild flu-like manifestations, which resolve entirely by the end of the disease to severe forms in a subset of patients, including severe pneumonia, acute respiratory distress syndrome, sepsis, thromboembolic manifestations, acute myopericarditis, septic shock, multi-organ failure, and eventually death **[1–4].** Although the exact pathogenic mechanisms behind the development of severe forms of the disease are not entirely explained yet, recent studies have highlighted the exaggeration of inflammatory processes and cytokine release, leading to cytokine storm and organ damage, in patients with extreme COVID-19 **[5].**

Previous studies have showed that the outbreak of COVID-19 is not equal among the different countries, with significant differences in the proportion of serious illnesses and mortality **[6].** While the adequacy of healthcare services may play a role in such inconsistencies, multicenter reports highlighted that patient-specific factors are significant determinants of the presentation and outcomes of COVID-19. Old age, male gender, comorbidities, and immune-compromised status were associated with severe presentations and poor outcomes **[7, 8];** besides, predisposing genetic factors may play a role in determining an individual’s susceptibility to infection and disease course as well **[9, 10].**

The Severe acute respiratory syndrome coronavirus-2 (SARS-CoV-2) enters the host cell by endocytosis after binding of its spike (S) protein to angiotensin-converting enzyme -2 (ACE-2) receptors in concert with S-protein priming by the host cell transmembrane serine protease TMPRSS. This leads to dysregulation of the angiotensin system and release of Tumor necrosis factor (TNF)-α along with interleukin (IL)-6 and other cytokine mediators, predisposing to the cytokine storm in severe COVID-19 **[11].** Age was found to be the most compelling risk factor of severe COVID-19 followed by pre-existing chronic medical illnesses, particularly multi-morbidity **[11, 12].** In addition, immune system remodeling, or immunosenescence that occurs in elderly patients, is considered the principal underlying factor for increased susceptibility to infection, particularly respiratory infections such as influenza and impaired immune responses to vaccination **[11].**

The innate and adaptive immune responses to viruses differ between males and females. The number and activity of cells of innate immunity, e.g., monocytes, dendritic cells, and inflammatory responses and type-I interferon production, are higher in females than males. In addition, toll-like receptor (TLR)-7 that detect single-stranded RNA viruses such as coronaviruses is encoded on the X chromosome with higher expression in females. Immune responses to viruses also vary with natural and induced changes in sex-hormone concentrations **[13].** Females also show higher humoral and cell-mediated immune responses to antigenic stimulation, vaccination, and infection compared to males **[13, 14].** Females have higher baseline immunoglobulin levels, antibody responses, B-cell gene expression, CD3+ and CD4+ cell counts, CD4+:CD8+ cell ratios, and T-helper cell (Th1), cytotoxic T-cell activity compared to males **[13, 14].** Sex steroids, e.g., testosterone, estradiol, and progesterone, influence cell signaling pathways and, consequently, immune cells’ functioning, leading to differential secretion of cytokines and chemokines **[13].**

Being a frequent tourism destination, international traffic, and a densely populated country, Egypt observed an increasing spread of COVID-19 since March 5; the official figures demonstrated that the number of confirmed cases was nearly 158,174 by 20 January 2021, with 8,696 deaths **[15].** Such mortality is notably higher than in other countries, which can be attributed to the potential role of patient-specific characteristics. Thus, we conducted the present retrospective study to assess the epidemiological, clinical and laboratory, characteristics associated with mortality among patients with COVID-19 from Egypt.

## Patients and methods

The present study was initiated after obtaining the protocol approval from the General Organization for Teaching Hospitals and Institutes (GOTHI) responsible ethics committees in Egypt. All patients signed a written informed consent on admission to share their data in research. The preparation of the present manuscript runs in compliance with the recommendations of the STROBE statement **[16].** All procedures performed in studies involving human participants were following the ethical standards of the institutional and/or national research committee and with the 1964 Helsinki Declaration and its later amendments or comparable ethical standards.

### Study design and population

The present study was a multicenter, retrospective study that retrieved the data of all Egyptian cases with confirmed COVID-19 from the electronic medical records of patients hospitalized at six hospitals affiliated to the General Organization For Teaching Hospitals and Institutes (GOTHI) through the period from the first of April to the end of July 2020. The included hospitals are Al-Sahel hospital, Al-Matareya hospital, Shebin hospital, Benha hospital, Damanhour hospital, and the National hepatology and tropical medicine research institute (NHTMRI). The diagnosis of COVID-19 was based on a positive reverse transcription-polymerase chain reaction (RT-PCR) laboratory test. Patients without or with negative RT-PCR results were excluded.

### Data collection and operational definitions

We retrieved epidemiological, clinical, laboratory, and radiological data of all eligible patients from the electronic medical records of the included hospitals. The epidemiological characteristics included age, gender, and smoking, while the clinical data included the COVID-19 symptoms and the associated comorbidities. The laboratory data included complete blood count (CBC) with differential count, C-reactive protein (CRP), serum ferritin, liver function tests, renal function tests, coagulation profile, D-dimer level.

Diabetes mellitus (DM) was defined as Hemoglobin A1C ≥6.5% or fasting plasma glucose ≥126 mg/dL or 2-hours plasma glucose ≥200 mg/dL in a patient with classic symptoms of hyperglycemia or hyperglycemic crisis, a random plasma glucose ≥200 mg/dl **[17].** Systemic hypertension was defined as grade I hypertension: systolic 140–159 mmHg, diastolic 90–99 mmHg, grade II hypertension: Systolic 160–179 mmHg, diastolic 100–109 mmHg, grade I hypertension: Systolic ≥180 mmHg, diastolic ≥110 mmHg **[18]**. Chronic kidney disease classification was based upon glomerular filtration rate (GFR) reduction (≥90 [grade 1, normal], 60–89 [grade 2, mildly decreased], 45–59 [grade 3a, mild to moderate decrease], 30–44 [grade 3b, moderate to severe decrease], 15–29 [grade 4, severe decrease], <15 [grade 5, kidney failure] ml/min/1.73 m2) **[19].** Glasgow Coma Scale (GCS) was categorized into severe (3–8), moderate (9–12), and mild (13–15) **[20].** Pulse rate (beats per minute (BPM)) was categorized into bradycardia (<60 BPM), Normal pulse rate (60–100 BPM), and tachycardia (>100 BPM). O2 saturation (%) was categorized into normal (95–100), mild hypoxemia (91–94), moderate hypoxemia (86–90), and severe hypoxemia (<85).

COVID-19 severity was categorized as mild, moderate, or severe according to the Egyptian Ministry of Health and Population management protocol. Mild cases included asymptomatic and symptomatic cases with lymphopenia (defined as an absolute lymphocyte count <1.0 × 103/L) or leukopenia (defined as a total leucocyte count <4.0 × 103/L) and no radiological evidence of pneumonia. Moderate cases included symptomatic patients with radiological features of pneumonia with or without leukopenia and lymphopenia. Severe and critical cases were defined by the presence of any of the following: SaO2 <92 without oxygen therapy; PaO2/FiO2 ratio <300 without oxygen or <200 with oxygen, chest radiology showing more than 50% lung involvement or progressive lung involvement within 24 to 48 hours. Severe and critical cases were indicated for intensive care unit (ICU) admission. Treatments were applied according to the protocol **[21].** The primary outcome of the study was in-hospital mortality of patients with confirmed COVID-19 cases.

### Statistical analysis

Data were analyzed using the software STATA 16 for Windows. Frequency counts and percentages summarized categorical variables. Continuous variables were represented as means ±standard deviations (SD) or median with interquartile range (IQR) according to data normality; the normality of the data was assessed by visual inspection of histograms and Shapiro-Wilk test. Comparison between moderate and severe cases to critically ill cases was made through univariate analysis as follows: categorical variables were assessed with the Chi-square test, whereas continuous variables were assessed using the Mann-Whitney test. The multivariate logistic regression model (forward stepwise selection) included all the variables with a p-value of <0.05 at the bivariate level. Variables, which achieved a p-value of <0.05 at the bivariate level, were grouped into the following four models: demographics, comorbidities, vital signs, and medications. Then, a generalized linear model (GLM) was reconstructed with covariates selected from the initial bivariate model based on the predictor’s statistical significance and clinically relevance. The odds ratio (OR) was calculated by using stepwise logistic regression modeling.

## Results

### Demographic characteristics

**Fig 1:**
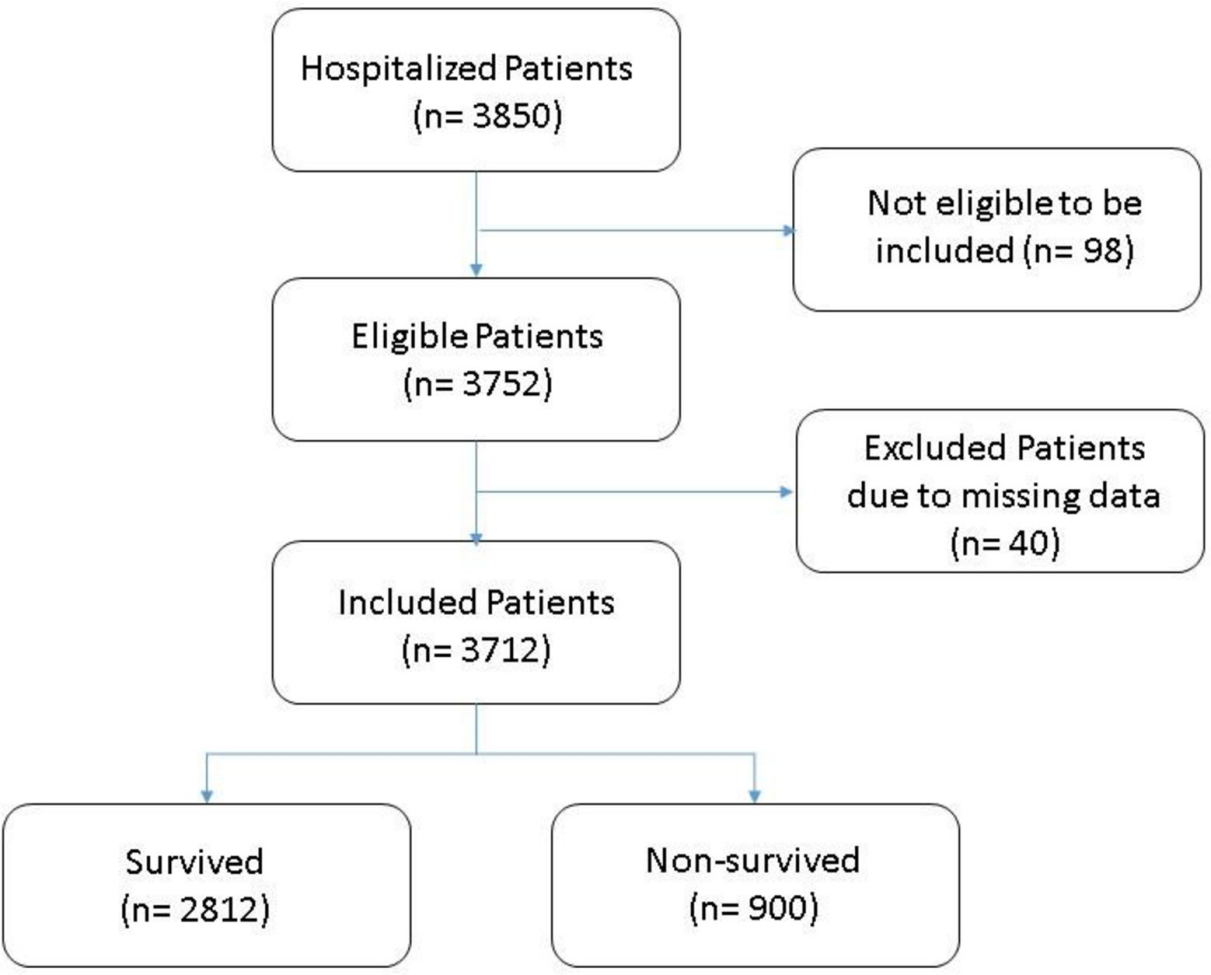
Flow chart of Egyptian COVID-19 patients included in the study.

**Fig 2:**
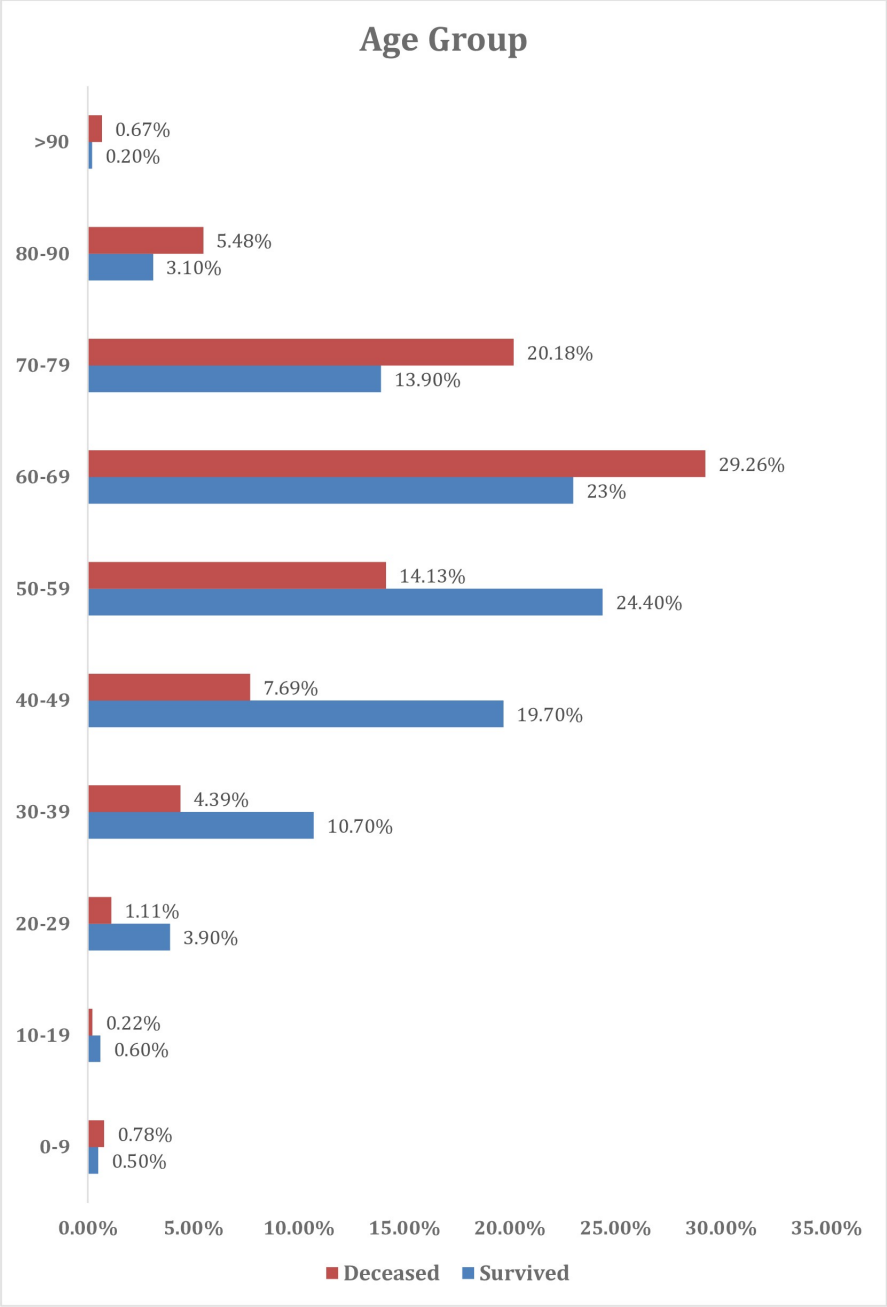
Baseline distribution of age groups.

The present cohort included 3712 hospitalized cases with COVID-19; of them, 900 deaths were recorded (24.2%). (**Fig 1)** The median days from hospital admission to death among those 900 cases was 6.1 days (range 0 to 97 days). Nearly 50% of the patients were males (n = 1837 patients) and patients aged ≥ 50 years old accounted for the majority of the sample (68.6%) (**Fig 2**). The most common symptoms of our cohort were Fever (56.88%), Cough (54.85%), dyspnea (34.60%), and fatigue (32.71%), with no significant difference between recovered and dead patients.

The mortality rate was higher among patients older than 60 years (65.7% versus 34.3% in patients younger than 60 years old, p-value <0.001). A total of 53.6% of deceased patients were males (p = 0.009), **Table 1**. Before death, 189 (21%) patients required mechanical ventilation. 87 (9.7%) patients required non-invasive ventilation (continuous positive airway pressure (CPAP)), of which ten patients progressed to invasive mechanical ventilation. None of the survived patients required mechanical ventilation.

**Table 1:** Demographic characteristics and comorbidities (n = 3712).

Variables	All (n = 3712)	Survived (n = 2812)	Deceased (n = 900)	P-value
**Age**				
0–9	21 (0.6)	14 (0.5)	7 (0.78)	**<0.001**
10–19	19 (0.5)	17 (0.6)	2 (0.22)	
20–29	121 (3.4)	110 (3.9)	11 (1.11)	
30–39	339 (9.5)	300 (10.7)	39 (4.39)	
40–49	623 (17.4)	554 (19.7)	69 (7.69)	
50–59	818 (22.9)	686 (24.4)	132 (14.13)	
60–69	911 (25.5)	647 (23)	264 (29.26)	
70–79	579 (16.2)	392 (13.9)	187 (20.18)	
80–90	135 (3.8)	87 (3.1)	48 (5.48)	
>90	11 (0.3)	5 (0.2)	6 (0.67)	
Missing	135 (3.63)	0	135 (15)	
**Gender**				
Male	1837 (49.9)	1364 (48.5)	473 (53.6)	**0.009**
Female	1875 (51.1)	1448 (51.5)	427 (46.4)	
**Comorbidities**				
Diabetes Mellitus	1155 (31.1)	817 (29.1)	338 (37.6)	**<0.0001**
Chronic Liver Diseases	143 (3.9)	116 (4.1)	27 (3)	0.127
Cerebrovascular Disease	35 (0.9)	13 (0.4)	22 (2.4)	0.35
malignancy	75 (2)	63 (2.2)	12 (1.3)	0.18
Hypertension	1106 (29.7)	771 (27.4)	335 (37.2)	**<0.0001**
Chronic renal Insufficiency	178 (4.1)	97 (3.4)	81 (9)	**<0.0001**
Ischemic heart disease	301 (8.1)	215 (7.6)	86 (9.6)	0.068
**Medications**				
Steroids	967 (26.1)	940 (33.4)	27 (3)	**<0.0001**
Therapeutic dose Anticoagulants	1470 (39.6)	1424 (50.6)	46 (5.1)	**<0.001**
Azithromycin	1719 (46.3)	1672 (59.5)	47 (5.2)	**<0.0001**
Hydroxychloroquine	238 (6.4)	238 (8.4)	0	**<0.001**
Chloroquine sulphate	38 (1)	38 (1.4)	0	**0.004**
Remdesivir	3 (0.001)	3 (0.001)	0	**0.32**
Oseltamivir	48 (1.3)	46 (1.6)	2 (2.2)	**0.001**
Nitazoxanide	19 (0.5)	19 (0.7)	0	**0.013**
Tocilizumab	4 (0.1)	4 (0.14)	0	**0.26**

Data are presented as number (percentage).

### Co-morbidities

Deceased patients had a significantly higher prevalence of diabetes than survived patients (37.6% versus 29.1% respectively; p-value <0.001). Besides, deceased patients were associated with higher prevalence of hypertension (37.2% vs. 27.4%, p-value <0.001) and chronic renal insufficiency (9% versus 3.4%, p-value <0.001), (**Table 1).**

### Vital signs and O_2_ saturation

Deceased patients had significantly higher pulse rate (p-value 0.003) and lower saturation rate (p-value <0.001) and GCS on admission (p <0.001). There was no significant difference between survived and no-survived patients in terms of systolic and diastolic blood pressure on admission (p-value 0.762 and p-value 0.577, respectively), (**Table 2)**.

**Table 2:** Baseline vital signs and laboratory parameters (n = 3712).

Variable	Survived (n = 2812)	Deceased (n = 900)	P-value
**Pulse rate, beats/mins,** Mean (SD)	90.01 (14.35)	91.98 (19.07)	**0.003**
**Tachycardia,** n (%)	697 (27.4%)	248 (36.4%)	**<0.0001**
**Hypoxemia,** n (%)	490 (34.9%)	561 (62.3%)	**<0.0001**
**O2 Saturation**	91 (88–95)	78 (70–89)	**<0.001**
**GCS,** Mean (SD)	14.71 (1.17)	12.52 (1.25)	**<0.0001**
**GCS <13,** n (%)	3 (0.1%)	218 (42.8%)	**<0.0001**
**Hemoglobin (mg/dL),** Mean (SD).	11.56 (1.116)	11.17 (2.2)	0.52
**White Blood Cells (10** ^**9**^ **cells/L),**	7.5 (5–11.5)	11 (6.1–14.6)	0.066
**Platelet count (x10** ^**6**^ **)**	192 (42–289)	173 (25–246)	**0.017**
**INR**	1 (1–1.37)	1.21 (1.21–1.7)	**0.011**
**Ferritin (ng/mL)**	25 (15–85)	44 (5–158.5)	**<0.0001**
**C-Reactive Protein (mg/L)**	0 (0–36)	48 (0–140)	**<0.0001**
**D-Dimer (ng/mL)**	500 (0–1250)	1267 (631–2041)	**<0.0001**
**Total bilirubin (mg/dL)**	0.9 (0.7–1)	1 (1–1.2)	0.122
**Direct bilirubin,** Mean (SD).	0.4 (0.3–0.6)	0.6 (0.3–1)	**0.015**
**Serum ALT (IU/dL)**	37 (22–48)	33 (15–53)	0.21
**Serum creatinine (mg/dL)**	0.67 (0.5–1)	1.2 (1–2.8)	**<0.001**

Data are presented as median (IQR), GCS: Glasgow coma scale, INR: International normalized ratio.

### Laboratory investigations

Deceased patients were associated with higher INR (p-value 0.01), serum creatinine (p-value <0.001), direct bilirubin (p-value 0.015), CRP (p-value <0.001), Serum ferritin (p-value 0.001), and D-dimer (p-value <0.001) at time of admission. In addition, deceased patients had lower platelet count (p-value 0.017), (**Table 2)**.

### Treatment

Deceased patients were less likely to receive azithromycin (p-value <0.001), anticoagulants (5.1% versus 50.6% in survived patients, p-value <0.001), and steroids (3% versus 33.4% in survived patients, p-value <0.001), (**Table 1)**.

### Univariate logistic regression

On univariate regression, we found that age >60 years (OR = 2.84, 95% CI (2.41–3.36, p <0.001), male gender (OR = 1.22, 95% 1.05–1.42, p = 0.009), diabetes mellitus (OR = 1.46, 95% CI 1.25–1.71, p <0.001), hypertension (OR = 1.57, 95% CI 1.33–1.83; p <0.001), and chronic renal insufficiency significantly increased the risk of death among admitted patients. Concerning vital signs, we found that tachycardia (OR = 1.52, 95% CI (1.27–1.81); p <0.0001), hypoxemia (OR = 1.24 (1.07–1.45); p = 0.006), and GCS <13 (OR = 701.5, 95% CI (223–2206); p <0.0001) significantly increased the risk of death among admitted patients (p = 0.001 and 0.03, respectively). Besides, higher CRP (p <0.001), serum ferritin (p <0.001), and D-dimer (p <0.001) at admission significantly increased the risk of death. The use of anticoagulants was associated with a reduced risk of mortality (OR 0.234, 95% CI 0.199–0.2760; p <0.001). Likewise, the use of azithromycin and steroids were associated with a reduced risk of death (**Table 3)**.

**Table 3:** Univariate risk predictors of COVID-19 mortality.

Variable	Deaths OR (95% CI)	P-value
**Age ≥ 60 years old**	2.84 (2.41–3.36)	**<0.0001**
**Male**	1.22 (1.05–1.42)	**0.009**
**Comorbidities**		
Diabetes Mellitus	1.46 (1.25–1.71)	**<0.0001**
Hypertension	1.57 (1.33–1.83)	**<0.0001**
Chronic renal Insufficiency	2.768 (2.04–3.756)	**<0.0001**
**Vital Signs**		
Tachycardia	1.517 (1.269–1.813)	**<0.0001**
Hypoxemia	1.241 (1.065–1.446)	**0.006**
GCS <13	701.5 (223–2206)	**<0.0001**
**Laboratory Parameters**		
Platelet (x10^6^)	0.999 (0.998–1.01)	0.07
INR	1.09 (1.014–1.172)	**0.02**
Ferritin (ng/mL)	1.002 (1.001–1.004)	**<0.0001**
CRP (mg/L)	1.008 (1.006–1.010)	**<0.0001**
D-Dimer (ng/mL)	1.3 (1.1–1.9)	**<0.0001**
Serum creatinine (mg/dL)	1.019 (1.005–1.034)	**0.008**
**Medications**		
Steroids	0.062 (0.042–0.091)	**<0.0001**
Anticoagulants	0.234 (0.199–0.2760)	**<0.001**
Azithromycin	0.038 (0.028–0.051)	**<0.0001**
Antivirals	0.176 (0.113–0.272)	**<0.0001**

CI: confidence interval, OR: odds ratio.

### Predictors of mortality

The GLM revealed that the following variables were independent positive predictors of mortality amongst hospitalized patients with COVID-19: age ≥ 60 years old (OR = 2.82, 95% CI 2.05–3.86; p <0.0001), diabetes mellitus (OR = 1.58, 95% CI 1.14–2.19; p = 0.006), hypertension (OR = 1.69, 95% CI 1.22–2.36; p = 0.002), chronic renal insufficiency (OR = 3.15, 95% CI 1.84–5.38; p <0.0001), tachycardia (OR = 1.65, 95% CI 1.22–2.23; p <0.001), hypoxemia (OR = 5.69, 95% CI 4.05–7.98; p <0.0001), and GCS <13 (OR 515.2, 95% CI 148.5–1786.9; p <0.0001). On the other hand, the use of therapeutic dose of anticoagulation (OR = 0.4, 95% CI 0.22–0.74, p = 0.003) and azithromycin (OR = 0.16, 95% CI 0.09–0.26; p <0.0001) were independent negative predictors of mortality (**Table 4**).

**Table 4:** Generalized linear nodels for risk predictors of COVID-19 mortality.

Variable	Deaths OR (95%CI)	P-value
Age ≥ 60 years old	2.82 (2.05–3.86)	**<0.0001**
Male	1.11 (0.83–1.49)	**0.49**
Diabetes Mellitus	1.58 (1.14–2.19)	**0.006**
Hypertension	1.69 (1.22–2.36)	**0.002**
Chronic renal Insufficiency	3.15 (1.84–5.38)	**<0.0001**
Tachycardia	1.65 (1.22–2.23)	**0.001**
Hypoxemia	5.69 (4.05–7.98)	**<0.0001**
GCS <13	515.2 (148.5–1786.9)	**<0.0001**
Steroids	0.52 (0.25–1.05)	**0.066**
Anticoagulants	0.4 (0.22–0.74)	**0.003**
Azithromycin	0.16 (0.09–0.26)	**<0.0001**
Antivirals	0.850 (0.508–1.421)	0.53

We also performed a multivariate analysis based on the following four models: 1) demographics and comorbidities; 2) vital signs; 3) laboratory investigations; and 4) treatment. Model 1 showed that age >60 years (OR 2.83, 95% CI 2.38–3.35, p = 0.001), male gender (OR = 1.211, 95% CI 1.1–1.43, p = 0.025), diabetes mellitus (OR 1.25, 95% CI 1.034–1.53, p = 0.022), hypertension (OR 1.51, 95% CI 1.243–1.84; p = 0.001), and chronic renal insufficiency (OR 3.398, 95% CI 2.45–4.71, p = 0.001) were independent predictors for mortality among admitted patients. Model 2 demonstrated that high pulse (OR = 1.012, 95% CI 1.006–1.019, p <0.001) and low GCS (OR = 1.003, 95% CI 1.001–1.005, p = 0.04) were significant predictors for mortality among admitted patients. None of the laboratory parameters on model 3 can be used as predictors for mortality. According to model 4, the use of therapeutic dose of anticoagulation medications was an independent predictor of mortality in hospitalized patients (OR 0.140, 95% CI 0.097–0201, p <0.001), **S1 Table.**

## Discussion

This study reports the outcome of hospitalized Egyptian patients with RT-PCR confirmed COVID-19 patients from 6 quarantine hospitals during the first peak of COVID-19 infection in Egypt (June-July 2020) **[22]**. The outcome of this study was the death of patients with positive RT-PCR for SARS-CoV-2. According to our final model, for patients older than 60 years, the odds ratio of COVD-19 mortality is expected to be higher by 127%. Similar to our study, older age was associated with increasing mortalities in studies including different populations **[23–25].** Data from China and Italy reported that the case fatality rate of COVID-19 significantly increases with age from ≤0.4% in patients aged in the 40s or younger to 14.8–220.2% in patients aged 80s or older. Data from France and the USA confirmed that hospitalizations and intensive care unit (ICU) admissions and mortality significantly increase with age **[11].** A large study by **O’Driscoll and colleagues, 2020** assessed the relationship between seroprevalence and the age-specific COVID-19-related mortality from 45 countries and 22 seroprevalence studies. They found that the COVID-19 infection fatality ratio is lowest among children aged 5–9-years, with a log-linear increase by age among individuals above 30 years **[26].**

Age-related alterations in innate and adaptive immunity against SARS-CoV-2 infection are still under investigation **[11].** The vulnerability of older adults to severe COVID-19 disease and death is largely related to immune system remodeling or immunosenescence and risk for immunopathology occurring in elderly patients with reduction of B and T lymphocyte functions **[11, 27].** Age-related change in innate and adaptive immunity is associated with impaired type-1 interferon (IFN) response. In addition, certain non-structural proteins of SARS-CoV-2 suppress the type-1 IFN response leading to suppressed CD8+ T-cell response to viral infection **[28].** It is hypothesized that age-related reduction of denovo T-cell response and/or impact from underlying medical illnesses, especially persistent viral infections, e.g., cytomegalovirus (CMV), could be a possible etiology of COVID-19 vulnerability in elderly patients **[11].** Older COVID-19 convalescent plasma donors were reported to have higher titers of SARS-CoV-2-specific IgG and neutralizing antibodies compared to younger donors, and the etiology is still unknown. **[11, 29, 30].** Inflammaging, chronic low-grade inflammatory phenotype (CLIP), persistent viral infection, e.g., CMV and other potential factors, e.g., smoking, reduced secretion of sex steroids and accumulated adipose tissue, leading to the unbalanced pro-inflammatory milieu in elderly adults, which potentiates further inflammatory reactions upon SARS-CoV-2 infection, and an exacerbated cytokine storm. It also influences ACE-2 expression and viral entry **[11, 31].**

Our findings showed male gender was associated wih higher risk of mortality compared to female (OR = 1.211, 95% CI: 1.1 to 1.43, p = 0.025). Similarly, **Nasiri and colleagues, 2020,** reported the male gender to be associated with mortalities from COVID-19 **[21].** Other cohorts from China, Italy, Denmark, and the USA also showed higher COVID-19 related mortalities in males **[24, 25, 32–34].** The underlying mechanisms could include sex chromosomes-related immunological response, different lifestyles that are higher in males (alcohol, smoking, lower rates of handwashing and obesity), and comorbidities **[33].**

A review by Gebhard et al., which studied the COVID-19 clinical and epidemiological gender differences from Europe and China, revealed no difference in the number of COVID-19 confirmed cases among males and females. However, the hospitalization, progression to severe disease, and case fatality rates were significantly higher in males. It was found that circulating ACE-2 is higher in healthy and diabetic males and in males with chronic kidney disease than females **[13].** Estrogens down-regulate type-1 angiotensin II receptor (AT2R) and regulate renin activity. Genes coding for ACE-2 and AT2R are located on the X chromosome, indicating a potential for higher expression in females **[13].** ACE-2 activity increased after Ovariectomy in females and decreased after orchiectomy in males **[13]**. TMPRSS2 is expressed significantly higher in the prostate than other body tissues; TMPRSS2 transcription is regulated by androgenic ligands and an androgen receptor binding element in the promoter. This may explain the high case fatality rate of COVID-19 in males **[35].** The X chromosome carries several immune-related genes, which may be variably expressed on both alleles in immune cells in females, influencing immune response. Oestradiol enhances T cell responses, neutrophil count, cytokine and antibody production, somatic hyper-mutation, and class switching. On the other hand, Testosterone suppresses the immune system **[36].** Our findings showed that pulse rate and O2 saturation are significant predictors of mortality in terms of vital signs, which is similar to the previously published literature.

Pre-existing chronic medical illnesses in patients with COVID-19 were frequently associated with increased disease severity and mortality. A study from the UK reported that higher mortality was associated with older age, male gender, cardiac disease, and pulmonary disease other than bronchial asthma, chronic renal insufficiencies, chronic hepatic disease, malignancy, and dementia **[37]. Harrison et al., 2020,** reported that ischemic heart disease or chronic renal insufficiencies are independent predictors of mortality in all age groups **[38].** A meta-analysis that included 1576 hospitalized patients in China reported that hypertension, chronic respiratory conditions, and cardiovascular disease are associated with severe COVID-19 **[39].** Our study did not show preexisting chronic liver disease as a factor associated with mortality. This is in contrast to other studies **[38, 40]. Singh and Khan, 2020** reported that underlying liver disease is associated with a high relative risk for mortality (RR, 2.8; 95% CI, 1.9–4.0; P-value <0.001) compared to patients without liver disease, and the relative risk showed further increase in patients with liver cirrhosis (RR, 4.6; 95% CI, 2.6–8.3; P-value <0.001) **[40].** This difference with our study may be related to the small number of patients with pre-existing liver disease in our cohort and a small number of patients with liver cirrhosis. **Boettler and colleagues, 2020** showed that patients with chronic liver disease did not show high prevalence among COVID-19 cases (<1%) **[41].** They suggested that patients with chronic liver disease are not at high risk of contracting SARS-CoV-2 infection. Besides, the risk of severe COVID-19 within such patients may depend on the underlying etiology of chronic liver disease and the stage of liver fibrosis and cirrhosis. **[41].** As regards that all our patients with chronic liver disease had underlying chronic viral hepatitis, mainly hepatitis C, **Boettler et al., 2020** also reported that little or no data has emerged to support that chronic viral hepatitis can influence the course of SARS-CoV-2 infection **[41].**

According to our final model, patients with DM will be expected to have a 52% increase in odds of dying from COVID-19. DM and the degree of hyperglycemia independently increase COVID-19 severity and mortality. Furthermore, the presence of DM-related complications, e.g., ischemic heart disease and chronic renal insufficiency, is associated with increased COVID-19 mortality **[42].** Patients with DM and COVID-19 develop disturbed glucose homeostasis, aggravation of inflammation, and immune system impairment. These conditions increase oxidative stress, cytokine release, and endothelial dysfunction, leading to increased liability for thromboembolism and organ damage. All these factors contribute to increased COVID-19 severity and rapid progression to cardiac and respiratory failure and consequently increased mortality **[42]**.

Our study found that the use of therapeutic dose anticoagulation, steroids, and azithromycin intake are independently associated with reduced mortality in our cohort. The significant difference between the frequency of administered treatments of each group could be explained by the more extended period of hospitalization in the survivors, which is associated with a higher frequency of treatment administration than with the non-survivors group who die between four to six days after hospitalization. Although contradictory data are present about the relation between anticoagulation and COVID-19 related mortality **[43],** the guidelines published by the European Society of Cardiology recommended the use of prophylactic enoxaparin (40 mg daily) for all patients hospitalized due to COVID-19 **[44].** The pathogenesis of hypercoagulable state that develops in patients with COVID-19 is not comprehensively understood. The triad of endothelial injury, stasis, and hypercoagulable state is involved (Virchow’s triad) **[45].** Studies comparing several doses of anticoagulants (from prophylactic to full therapeutic doses) are very few, and further studies are still needed **[45].** Limitations of this study include its retrospective nature and retrieval of data from electronic medical records that some details of comorbidities might not be complete. Another limitation is the missing of some valuable data, including the respiratory rate and exact age of the included patients. In addition, we could not perform a subgroup analysis based on the study’s centers, due to the lack of relevant data. The lack of detailed medical records of patients is a possible data, and methodological bias and the lack of previous influenza or respiratory infection vaccination records are unadjusted potential cofounders.

In conclusion, this study shows that Age above 60 years, male gender, and comorbidities, including the presence of DM, hypertension, and chronic renal insufficiency, On-admission high pulse rate, and low Glasgow coma scale are independent predictors of mortality in hospitalized Egyptian patients with COVID-19. The use of anticoagulants, Steroids, and azithromycin is associated with reduced mortality in Egyptian patients with COVID-19, and further studies are needed to confirm the benefits of these medications in patients with COVID-19. The study described potential predictors of mortality amongst COVID-19 patients, including demographics, clinical features, laboratory data, and received treatment. We did not intend to explore the predictors in different age/gender groups. We believe that the proposed subgroup analyses would weaken the robustness of our data. Thus, we recommended future studies to explore potential predictors in different age/gender groups.

## Supporting information

S1 Table Multivariate risk predictors of COVID-19 mortality. (DOCX)
