# Supplementary material for: Prediction of mortality in hospitalized Egyptian patients with Coronavirus disease-2019: A multicenter retrospective study
Source: PLoS One. 2022 Jan 11;17(1):e0262348. doi: 10.1371/journal.pone.0262348 (PMC8752027; doi:10.1371/journal.pone.0262348)
Supplement: S1 Table — (DOCX) [file pone.0262348.s001.docx]

**S1 Table:** **Multivariate risk predictors of COVID-19 mortality**

| **Variable** | **Deaths**  **OR (95%CI)** | **P-value** |
| --- | --- | --- |
| Age ≥ 60 years old | 2.83 (2.38 - 3.35) | **<0.0001** |
| Male | 1.211 (1.1 - 1.43) | **0.025** |
| **Comorbidities** | | |
| Diabetes Mellitus | 1.25 (1.034 - 1.53) | **0.022** |
| Hypertension | 1.51 (1.243 - 1.84) | **<0.0001** |
| Chronic renal Insufficiency | 3.398 (2.45 - 4.71) | **<0.0001** |
| **Vital Signs** | | |
| Tachycardia | 1.97 (1.49 - 2.59) | **<0.0001** |
| Hypoxemia | 8.37 (6.12 - 11.47) | **<0.0001** |
| GCS <13 | 393.84 (123.18 - 1259.24) | **<0.0001** |
| **Laboratory data** | | |
| INR | 1.199 (0.564 - 2.548) | 0.63 |
| Ferritin (ng/mL) | 0.872 (0.781, 1.073) | 0.15 |
| CRP (mg/L) | 0.985 (0.956 - 1.014) | 0.29 |
| D-Dimer | 1 (1 – 1) | 0.16 |
| Serum creatinine (mg/dL) | 1.39 (0.79 - 2.453) | 0.24 |
| **Medications** | | |
| Steroids | 0.397, (0.247 - 0.639) | **<0.0001** |
| Anticoagulants | 0.140, (0.097 – 0201) | **<0.001** |
| Azithromycin | 0.038, (0.028 - 0.051) | **<0.0001** |
| Antivirals | 0.850, (0.508 - 1.421) | 0.53 |
